# Diagnostic Limitations, Patient Characteristics, and Confounding Factors Impacting Neurotologic Lesion Imaging: A Systematic Review

**DOI:** 10.3390/diagnostics16030446

**Published:** 2026-02-01

**Authors:** Diana Hamdan, Precious Ochuwa Imokhai, Alexandra Konvalina, BaoKhanh Nguyen, Maha Alhoda, Valentina Alejandra Da Silva Acosta, Waseem Syed, Amanda Brooks

**Affiliations:** 1 College of Osteopathic Medicine, Kansas City University, Joplin, MO 64804, USA; 2 Montana College of Osteopathic Medicine, Rocky Vista University, Billings, MT 59106, USA; 3 School of Osteopathic Medicine, A.T. Still University, Mesa, AZ 85206, USA; 4 School of Medicine, Royal College of Surgeons in Ireland, Muharraq 228, Bahrain; 5 Faculty of Health Sciences, Universidad Anáhuac México Norte, Mexico City 52786, Mexico; 6 College of Osteopathic Medicine, Lake Erie College of Osteopathic Medicine, Bradenton, FL 34211, USA; 7 Department of Research in Scholarly Activity, College of Osteopathic Medicine, Rocky Vista University, Ivins, UT 84738, USA

**Keywords:** neurotologic lesions, diagnostic imaging, MRI and CT accuracy, socioeconomic disparities, interpretation bias

## Abstract

**Background**: Neuroimaging protocols for neurotologic disease are often developed without consideration of patient-specific factors such as biological differences, clinical presentation variability, and comorbidities. This lack of tailored design contributes to insufficient detection, delayed diagnosis, and inappropriate treatment. **Objectives**: To critically examine the literature on diagnostic limitations of neuroimaging for neurotologic lesions and identify gaps in protocol validation, accuracy, and clinical translation. **Methods**: A systematic review of PubMed and Google Scholar was conducted, focusing on studies published between 2015 and 2025 that evaluated diagnostic imaging outcomes in patients with neurotologic lesions. Eligible studies included prospective cohorts, retrospective analyses, and consensus statements. Outcomes of interest included the sensitivity and specificity of imaging modalities, prevalence of misdiagnosis, and the influence of biological, anatomical, and clinical variability on diagnostic performance. **Results**: The literature demonstrates that neurotologic disorders are frequently associated with diagnostic challenges, including atypical clinical presentations, overlapping symptoms, and stroke mimics, which complicate image interpretation. Standard magnetic resonance imaging (MRI) protocols often miss subtle or early ischemic changes, resulting in delayed intervention. Few studies stratify outcomes by patient characteristics, and most protocols were developed in generalized populations without comprehensive validation. Evidence on advanced imaging modalities (positron emission tomography (PET), single-photon emission computed tomography (SPECT), high-resolution MRI) remains limited, and large-scale prospective studies addressing diagnostic accuracy gaps are lacking. In summary, a total of 27 studies met inclusion criteria. **Conclusions**: Current neuroimaging methods are insufficiently validated across diverse patient populations, contributing to the underdiagnosis and mismanagement of neurotologic disease. Improved diagnostic accuracy will require large-scale, prospective research, standardized outcome reporting, and imaging protocols designed to account for patient-specific variability.

## 1. Introduction

Neurotologic lesions comprise different underlying pathologies related to the tissues and structures of the ear, vestibular system, skull base, and temporal bone [1,2]. The etiology of neurotologic lesions can be infectious, inflammatory, traumatic, congenital, or neoplastic [3]. Patients with neurotologic lesions may exhibit symptoms including hearing loss, vertigo, facial weakness, and tinnitus [4]. Neurotologic lesions represent a significant health concern, with an 8.8 per 1000 prevalence of dizziness-related ambulatory visits in the United States (US) from 2013–2015, with follow-up imaging ordered in 5.5% of cases [5].

Timely assessment and diagnosis of neurotologic lesions is critical to improving patient outcomes, lowering morbidity, irreversible damage, and the burden of additional interventions, especially when the underlying condition is benign. The two most common imaging techniques used in the treatment of neurotologic lesions include magnetic resonance imaging (MRI) and computed tomography (CT) [2]. Since its introduction, MRI has remained an essential tool in the identification and treatment of neurotologic lesions as the technology has improved over time. MRI is widely regarded as the gold standard for differentiating soft-tissue lesions of the middle ear, particularly with the use of diffusion-weighted imaging (DWI), and is commonly used in the diagnosis of acoustic neuroma (vestibular schwannoma) [6]. Nonetheless, MRI may offer limited delineation of fine anatomic detail, and its spatial accuracy can be affected by motion and sequence-related artifacts [7].

Computed tomography (CT) has long been first-line for identifying neurotologic lesions due to its excellent spatial resolution for bony anatomy, but its specificity is limited when differentiating soft tissue lesions from inflammatory tissue [8]. Combining MRI and CT allows both techniques to complement each other, providing the ability to contrast soft and dense tissue features [9]. Both cone beam CT and non-echo planar diffusion-weighted (Non-EPI DWI) MRI are used extensively in the identification of lesions such as congenital cholesteatoma [1]. The diagnosis landscape has continued to evolve with emerging technologies, including positron emission tomography (PET) and more advanced MRI techniques, such as DWI-MRI [10,11] and delayed three-dimensional fluid-attenuated inversion recovery MRI (delayed 3D-FLAIR MRI) [12].

While these imaging techniques are vital tools in a physician’s arsenal for diagnosing neurotologic conditions, there are inherent limitations for each technique that need to be considered. Imaging modalities are limited for the diagnosis of inflammatory neurotologic disorders, such as vestibular neuritis, which can appear normal under MRI even with contrast-enhancing techniques [2]. For instance, a case study of 15 patients with resected internal auditory canal tumors demonstrated the limitations of imaging as a diagnostic technique, where MRI was unable to provide a clear diagnosis in the majority of cases [13]. From a population health perspective, access to MRI remains an ongoing concern, particularly in medically underserved communities [14]. This challenge is compounded by limited validation of imaging performance across diverse patient populations, which may increase the risk of diagnostic error or misinterpretation, especially for subtle neurotologic phenotypes. Consequently, opportunities remain to improve the specificity, equity, and generalizability of commonly used imaging techniques.

While previous reports have discussed the use of imaging modalities in selected neurotologic conditions, a comprehensive evaluation of diagnostic accuracy limitations and population-level challenges across imaging techniques remains to be addressed. This systematic review aims to evaluate diagnostic limitations of current neuroimaging modalities for neurotologic lesions and to examine how patient characteristics, socioeconomic factors, and radiologist expertise influence diagnostic accuracy. This review is intended to provide clinically actionable guidance for radiologists, otolaryngologists, neurologists, and referring physicians to improve imaging selection, interpretation, and patient outcomes.

## 2. Materials and Methods

This systematic review followed Preferred Reporting Items for Systematic Reviews and Meta-Analyses (PRISMA) 2020 (Table S1). Because only published, aggregate-level data were used, institutional review board approval was not required.

### 2.1. Search Strategy

For this systematic review, two independent reviewers conducted a search of the PubMed database using combinations of terms related to neurotologic lesions, neuroimaging modalities (MRI, CT, PET, and SPECT), and diagnostic limitations. The Boolean search string, formatted for PubMed, is provided below (the [tiab:~0] operator denotes a string-literal search):

((schwannoma [All Fields] OR “otitis externa” [All Fields] OR “paraganglioma” [All Fields] OR cholesteatoma [All Fields] OR otosclerosis [All Fields]) AND (“diagnostic accuracy” [tiab:~0] OR “diagnostic sensitivity” [tiab:~0] OR “false results” [tiab:~0] OR “interobserver agreement” [tiab:~0] OR socioeconomic status* [All Fields] OR “racial differences” [All Fields] OR “socioeconomic determinant*” [All Fields] OR “imaging in management of” [tiab:~0] OR “complementary roles of” [tiab:~0] OR “imaging evaluation in” [tiab:~0] OR “disparities in management” [tiab:~0] OR “target volume definition” [tiab:~1] OR “gold standard for baseline imaging” [tiab:~1] OR “economic evaluation” [All Fields])) OR (“MRI CT machine accessibility” [tiab:~2] OR (“Medical image interpretation” [All Fields] AND “Medical image perception” [All Fields]) OR “diagnostic imaging errors” [All Fields] OR “imaging for otolaryngologic” [tiab:~0]).

### 2.2. Inclusion and Exclusion Criteria

We screened articles published between 2015 and 2025 that evaluated the diagnostic performance of neuroimaging—reporting outcomes such as sensitivity, specificity, false positives, false negatives, or diagnostic limitations—involving patient populations across all ages, ethnicities, geographic regions, and socio-economic backgrounds. Review articles, articles with non-human subjects, and studies published in a language other than English were excluded. Observational and diagnostic accuracy studies were eligible for inclusion, without restriction to a specific study design.

In the first phase of article selection, two reviewers independently evaluated the titles and abstracts of articles that resulted from the initial search. Subsequently, articles considered relevant for this investigation were subjected to a review of the entire text. Final inclusion was determined based on consensus between both reviewers.

To minimize the risk of bias during the screening process, article screening and selection were conducted independently and manually by the two research team members. This approach is intended to ensure objectivity and consistency in the selection and evaluation of studies included in the study.

## 3. Results

### 3.1. Study Screening and Selection

**Figure 1:**
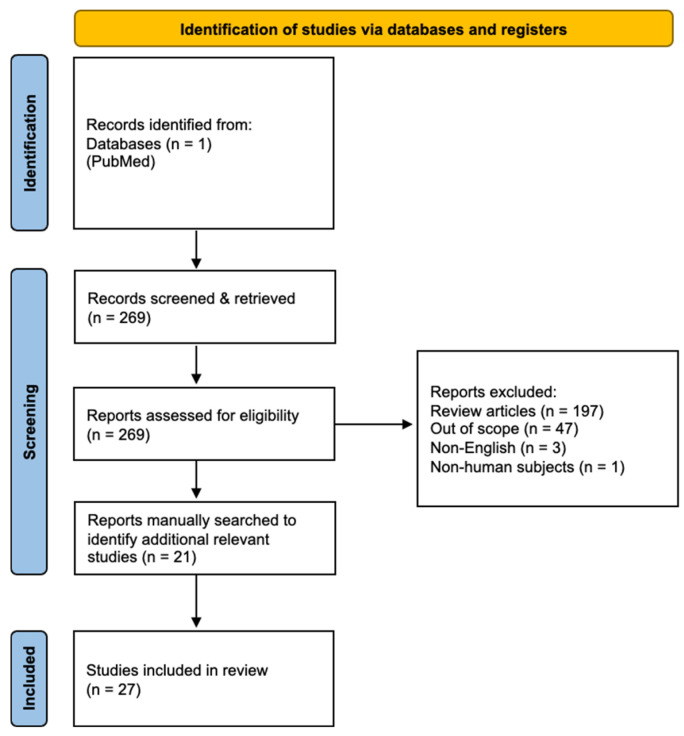
PRISMA flow diagram illustrating study selection (27 studies included).

The initial screen identified 269 studies (Figure 1). Of these, articles were excluded for the following reasons: systematic reviews (*n* = 197), studies not primarily reporting imaging outcomes (n = 47), non-English publications (n = 3), and non-human studies (n = 1), leaving 21 articles for inclusion. Reference lists of these articles were manually screened, yielding six additional reports.

Studies were included based strictly on predefined eligibility criteria and not on perceived importance. Inclusion required reporting of diagnostic performance metrics (e.g., sensitivity, specificity, false results). Two reviewers independently screened titles/abstracts, followed by full-text review, with discrepancies resolved by consensus. Included studies were categorized as either (1) diagnostic accuracy studies reporting sensitivity, specificity, or false results, or (2) health services studies evaluating socioeconomic and access-related imaging disparities. Table 1 summarizes key study characteristics and outcomes of the included articles.

**Table 1:** A summary of key study characteristics.

Article Reference	First Author (Year)	Country	Study Design	Population (n)	Inclusion Criteria	Exclusion Criteria	Intervention/Exposure	Comparator	Primary Outcome(s)	Key Results
[15]	Yigiter et al. (2015)	Turkey	Prospective cohort study	54	Patients with suspected middle ear cholesteatoma on clinical examination		EP-DWI	High resolution computed tomography (HRCT)	Diagnostic values	EP-DWI is a reliable technique for the imaging of cholesteatoma
[16]	Pietraszek et al. (2025)	Poland	Retrospective cohort study	156	Patients who were diagnosed with cholesteatoma and underwent surgical treatment		DWI non-EPI	Intraoperative findings	Concordance of clinical and radiological findings	MRI DWI non-EPI is an effective imaging technique for detection of cholesteatoma recurrence
[10]	Piekarek et al. (2022)	Poland	Retrospective cohort study	32	Patients with suspected cholesteatoma who underwent MRI of the temporal bone		DWI non-EPI	EPI-DWI	Sensitivity, specificity, and intraobserver agreement	Non-EPI DWI is a reliable technique for the detection of cholesteatoma
[11]	Benson et al. (2021)	USA	Retrospective cohort study	23	Patients who had preoperative MRI including Half-Fourier Acquisition Single-shot Turbo Spin Echo (HASTE) and RESOLVE sequences for evaluation of cholesteatoma and subsequent operation in which diagnosis was confirmed	Images degraded by artifacts	HASTE imaging protocol	RESOLVE imaging protocol	Detection of primary and recidivistic cholesteatoma	HASTE outperformed RESOLVE in detection of cholesteatoma
[17]	Locketz et al. (2016)	USA	Case series	12	Adults with preoperative temporal bone CT and PROPELLER DW-MRI scans who underwent cholesteatoma surgery		PROPELLER DW-MRI	CT, DW-MRI	Diagnostic and localization accuracy	CT and PROPELLER DW-MRI fusion can improve identification and localization of disease
[18]	Pizzini et al. (2020)	Italy	Retrospective cohort study	66	Patients with a histological diagnosis of vestibular schwannoma and MRI studies with both sequences (enhanced T1-WI and HRT2-WI)		HRT2-WI	Gd T1-WI	Diagnostic accuracy in measuring the size of vestibular schwannoma	Gadolinium can be safely omitted only in surveillance studies of vestibular schwannoma.
[19]	Coelho et al. (2018)	United States	Retrospective cohort study	50	Patients with previous diagnosis of vestibular schwannoma, available T1-weighted contrast-enhanced MRI studies and HRT2-weighted imaging, post-diagnostic imaging follow-up, and treated in the VCU health system between January 1998 and May 2011.	Neurofibromatosis type 2. History of previous surgery or radiotherapy. MRI studies without availability of both sequences (T1C and HRT2).	High-resolution T2-weighted (HRT2) MRI	Contrast-enhanced T1-weighted (T1C) MRI	Consistency and accuracy in measuring vestibular schwannoma size. Determining whether both techniques are clinically equivalent for surveillance.	High-resolution T2-weighted MRI without contrast is a reliable and less cost-effective alternative for monitoring patients with known vestibular schwannoma. Routine use of gadolinium is not required in follow-up studies.
[20]	Tolisano et al. (2019)	United States	Case series	23	Patients with diagnosis of vestibular schwannoma under surveillance or treated with radiotherapy. At least two MRI studies with simultaneous acquisition of T1 with contrast (T1C) and high-resolution T2 (HRT2).	Neurofibromatosis type 2. MRI studies without availability of both sequences (T1C and HRT2).	High-resolution T2-weighted (HRT2) MRI	Contrast-enhanced T1-weighted (T1C) MRI	Correlation between linear and volumetric measurements of vestibular schwannoma in T1C vs. HRT2. Consistency in the interpretation of tumor growth between sequences and between observers.	Although T1C and HRT2 show high consistency and reproducibility, volumetric measurements on contrast-enhanced T1-weighted images may be more reliable for assessing vestibular schwannoma growth. Linear measurements are highly reproducible in both sequences.
[21]	Forgues et al. (2018)	United States	Retrospective cohort study	26	Adult patients with diagnosis of vestibular acoustic neuroma/schwannoma and at least three MRI studies of the internal auditory canal. Studies obtained between 1 January 2008 and 11 October 2016.	Bilateral vestibular schwannomas. Other intracranial tumors. Neurofibromatosis type 2. Post-surgical studies in surgical patients. Patients with <3 eligible MRI studies.	High-resolution T2-weighted (HRT2) MRI	T1-weighted post-contrast MRI	Accuracy of non-contrast T2 MRI for tumor size measurement and identification of acoustic neuroma growth.	Non-contrast T2-weighted MRI is reasonably accurate for measuring size and detecting growth of vestibular schwannomas. Gadolinium is not essential for all follow-up examinations, but it remains useful in selected cases.
[22]	de Bresser et al. (2024)	The Netherlands	Single-center pilot study	25	Patients evaluated between 2016 and 2023 with clinical or genetic suspicion of head and neck paragangliomas carrying HNPGL-associated genetic variants with a head and neck MRI and [68Ga]Ga-DOTATOC PET/CT performed within an interval of ≤12 months.		[68Ga]Ga-DOTATOC PET/CT	Head and neck MRI	Location of head and neck paragangliomas	The authors recommend changing the gold standard for baseline MRI/CT imaging to [68Ga]Ga-DOTATOC PET/CT (when available). MRI/CT would then be used as a follow-up tool, not for initial staging, especially in carriers of genetic variants.
[23]	Ueda et al. (2025)	Japan	Case report	3	Patients with suspected or confirmed jugulotympanic paragangliomas who underwent CT, MRI, and 68Ga-DOTATOC PET imaging for diagnostic evaluation and treatment planning.	Patients not undergoing multimodal imaging, those with non-paraganglioma temporal bone lesions, or cases lacking sufficient clinical or imaging data for diagnostic assessment	CT/MRI + 68Ga-DOTATOC PET	CT/MRI without PET	Impact of 68Ga-DOTATOC PET on diagnostic confirmation and treatment decision-making in patients with jugulotympanic paragangliomas when added to CT and MRI	68Ga-DOTATOC PET serves as a valuable adjunct diagnostic imaging to CT and MRI for evaluating jugulotympanic paragangliomas, confirming diagnosis, and guide treatment planning
[24]	Maurice et al. (2012)	Germany	Retrospective cohort study	15	Patients with known phaeochromocytoma or paraganglioma (PCC/PGL) who underwent both 68Ga-DOTATATE PET/CT and 123I-MIBG SPECT imaging within 6 months without interval treatment changes	Patients without paired imaging	68Ga-DOTATATE PET/CT used for detection and follow-up of PCC/PGL lesions	123I-MIBG SPECT scintigraphy (with CT/MRI as supportive anatomical imaging)	Lesion detection sensitivity of 68Ga-DOTATATE PET/CT vs. ^123^I-MIBG SPECT on both a per-patient and per-lesion basis	68Ga-DOTATATE PET/CT detected more lesions than 123I-MIBG across all anatomical sites, especially bone and head/neck lesions, and omission of DOTATATE would have adversely affected management supporting its preferential use in high-risk or metastatic PCC/PGL
[25]	Kim et al. (2023)	Brazil	Systematic review/Meta-analysis	37	Studies with more than 10 patients diagnosed with necrotizing otitis externa that reported diagnostic sensitivity data for radiologic imaging modalities	Case reports, review articles, non-English studies, and studies lacking diagnostic imaging accuracy data	CT, MRI, technetium-99m, and gallium-67	Other imaging modalities or diagnostic criteria within the same patient population	Diagnostic sensitivity of each imaging modality for detecting necrotizing otitis externa	Technetium scans, gallium scans, and MRI are sensitive for diagnosing NOE, and CT works better when both bone and soft-tissue changes are considered, but imaging alone is not specific and clear diagnostic criteria are still needed
[26]	Haleem et al. (2025)	USA	Retrospective cohort study	33	Adults admitted with suspected necrotizing otitis media defined by persistent otalgia/otorrhoea plus ≥2 risk factors	Patients with insufficient clinical suspicion or an alternative diagnosis	Initial CT followed by technetium bone scintigraphy or MRI when CT was negative	CT-based evaluation without nuclear medicine imaging	The effectiveness of the departmental NOE diagnostic and treatment algorithm and assess adherence to established protocols	The study concluded that a standardized necrotizing otitis media management algorithm, using technetium bone scans when CT is inconclusive alongside clinical assessment, improves diagnostic accuracy and treatment success
[27]	Daqqaq et al. (2021)	Saudi Arabia	Retrospective cohort study	51	Patients with bilateral profound sensory neural hearing loss (SNHL)	Not specified	MDCT and MRI	Preoperative evaluation of cochlear implant candidates	Comparison of imaging findings with patient health history and other data	MDCT was superior in the demonstration of middle ear disease while MRI was more useful in the delineation of the cochlear nerve and cochlear patency
[28]	Bassiouni et al. (2023)	Germany	Retrospective chart review	40	Patients undergoing high-resolution CT of the temporal bone for suspected otosclerosis	Not specified	HRCT	HRCT images reviewed retrospectively by an experienced neuroradiologist who was blinded to the initial report.	Initial radiological diagnosis vs. re-evaluation	A substantial proportion of otosclerosis cases were missed on initial CT interpretation, highlighting diagnostic pitfalls and reporting limitations.
[29]	Berrettini et al. (2010)	Italy	Diagnostic Accuracy Study	45	Patients with clinical suspicion of otosclerosis	Not specified	SPECT and CT imaging	SPECT vs. CT	Diagnostic performance of imaging modalities in otosclerosis	Combined SPECT and CT improved detection and assessment of otosclerotic foci compared with CT alone.
[30]	Vagal et al. (2017)	USA	Retrospective cohort study	Not specified	Patients presenting with acute stroke	Not specified	Use of neuroimaging (CT, MRI)	Age, sex, and racial subgroups	Differences in neuroimaging utilization	Significant disparities in neuroimaging use were observed by age, sex, and race in acute stroke care.
[31]	Wang et al. (2024)	USA	Retrospective cohort study	Not specified	Patients presenting with acute stroke	Not specified	Acute stroke imaging utilization	Gender (male vs. female)	Imaging use, treatment rates, and clinical outcomes	Gender-based disparities in imaging utilization were identified, with downstream effects on treatment and outcomes.
[32]	Wang et al. (2022)	USA	Retrospective cohort study	85,547 stroke episodes	Patients presenting with ischemic stroke	Patients with TIA or intracranial hemorrhage	Use of neuroimaging	Race, sex, age, income, stroke severity	Trends in neuroimaging use, image to treatment associations, mortality outcomes	CTA/CTP use increased, MRI slight rise, MRA declined. Black, female older patients from rural areas received less advanced imaging. CTA/CTP use correlated with higher IVT/EVT rates. CTA, MRI and MRA associated with lower mortality.
[33]	Khalid et al. (2022)	Pakistan	Cross-sectional study	148	Patients presenting with schwannoma	Not specified	Surgical management of schwannoma	Socioeconomic class subgroups, Hospital type and volume, Age groups, Extent of surgery	Surgical incidence and prevalence of schwannoma, demographic distribution, treatment characteristics	Schwannomas consisted of a small percentage of national brain tumor cases. Short term mortality was higher compared to higher income country benchmarks, follow up completion was limited.
[34]	Brinjikji et al. (2014)	USA	Retrospective cohort study	210,212	Hospital admission with primary ICD-9 codes for acute ischemic stroke (433.x1, 434.x1), Admission classified as urgent/emergent, Imaging and billing data available during index hospitalization	Transfer patients (to avoid missing outside imaging), Non-primary stroke diagnoses and non-urgent/elective admissions	Insurance status (uninsured, Medicaid, Medicare, private insurance)	Private-insurance patients vs. all other insurance categories	Utilization of imaging during hospitalization like Head CT, perfusion CT, Head MRI, Noninvasive head/neck angiography (CTA/MRA), Carotid Ultrasound, Echocardiography	Significant disparities persisted after adjustment for age, sex, race, and hospital factors. Compared with privately insured patients, odds of receiving advanced imaging were lower: Uninsured: head MRI OR 0.77; head angiography OR 0.78; neck angiography OR 0.79; Medicaid: head MRI OR 0.64; head angiography OR 0.67; neck angiography OR 0.67; Medicare: head MRI OR 0.41; head angiography OR 0.76; neck angiography OR 0.82. Echocardiography and carotid ultrasound were also less frequently used in Medicaid/Medicare groups. No major disparities were seen for perfusion CT (likely triage-driven). The authors concluded that insurance-based disparities in stroke imaging may influence treatment opportunities and outcomes, and call for system-level investigation.
[35]	Wang et al. (2022)	USA	Retrospective cohort study	24,487	Laboratory-confirmed COVID-19 infection and hospital admission with available clinical and imaging variables needed for model evaluation	Missing key outcome variables, Incomplete predictor data required for model testing, Pediatric patients	Deep-learning mortality prediction models using clinical and imaging features	Performance of the same models when transported across independent U.S. health systems (external validation) vs. original development performance	In hospital mortality and model performance metrics (AUC, calibration, sensitivity/specificity) across sites.	Models that performed well in original institutions lost accuracy and calibration when applied to new hospitals. Variability in demographics, comorbidities, treatment patterns, and imaging practices significantly reduced generalizability. Site-specific retraining or recalibration improved performance but did not fully eliminate bias. The authors warned against deploying “off-the-shelf” AI models in clinical care without rigorous local validation.
[36]	Zeitouni et al. (2024)	USA	Retrospective cohort study	482	Patients attending otolaryngology clinical visits	Not specified	Demographic and socioeconomic factors	Different demographic and socioeconomic groups	Rates of missing laboratory tests and imaging	Missing labs and imaging were associated with demographic and socioeconomic determinants, indicating inequities in access and follow-through.
[37]	Carlson et al. (2016)	USA	Retrospective cohort study	9782	Patients diagnosed with vestibular schwannoma in Surveillance, Epidemiology, and End Results (SEER) (2004–2012)	Bilateral VS, cases with neurofibromatosis, NOS	exposure is race/ethnic categories	Between race comparisons of vs. presentation, management, and survival	annual incidence of vestibular schwannoma across racial groups; tumor size at diagnosis; treatment modality distribution (observation, radiation, microsurgery); overall survival across race groups; adjusted odds of receiving surgery after controlling for tumor size, age, and treatment center.	white patients had the highest annual incidence rates, whereas black and Hispanic groups had the lowest. black, Hispanic, and Asian patients presented with larger tumors at initial diagnosis. after adjustment, Hispanic patients were more likely than white patients to undergo microsurgical treatment. overall survival did not differ significantly across all races collectively, but in the postoperative subgroup, black and Hispanic patients demonstrated worse survival compared with white patients. findings indicate meaningful racial differences in presentation, management selection, and outcomes.
[38]	Pandrangi et al. (2020)	USA	Retrospective cohort study	14,507	Patients with SEER recorded cases coded as vs. using ICD-O-3 histology code 9560/0	Not specified	Patient demographic and tumor factors	demographic and tumor groups	Age-adjusted incidence, tumor size distribution, treatment modality patterns, demographic predictors of tumor size and treatment, overall survival	Incidence stable at 1.4/100k; highest in older adults; younger age and Asian/Pacific Islander race associated with larger tumors; older age predicted observation/radiation; younger age and larger tumors predicted surgery; Black and American Indian/Alaskan Native patients more likely to undergo observation and less likely to receive surgery; tumor size strongly correlated with operative management
[39]	Mullins et al. (2002)	USA	Retrospective cohort study	733	Patients admitted with suspected early stroke who had unenhanced CT or diffusion-weighted MRI at admission, and a definitive discharge diagnosis confirming stroke vs. no stroke.	Missing records; Diagnosis of transient ischemic attack; Imaging performed after intra-arterial thrombolysis; Lack of definitive discharge diagnosis	Availability vs. absence of clinical history indicating suspected early stroke when interpreting imaging.	Same imaging interpreted without explicit clinical suspicion of stroke.	Sensitivity, specificity, predictive values, and accuracy of unenhanced CT, diffusion-weighted MRI for detecting early stroke	Unenhanced CT: Sensitivity improved when clinical history indicated stroke (52% vs. 38%, *p* = 0.008) and Specificity remained high (96% vs. 89%) Diffusion-weighted MRI: Sensitivity remained high regardless of clinical history (95% vs. 94%) and Specificity similarly high (95–98%)
[40]	Oray et al. (2015)	Turkey	Retrospective cohort study	50	Patients who were submitted to DW-MRI for a suspected acute ischemic stroke	Not specified	Different physician observers	Emergency Physicians	Inter-observer agreement between reviewers	Diffused-weighted MRI is an effective tool for detecting ischemic stroke, across multiple physician interpreters
[41]	Treviño et al. (2021)	USA	Commentary	Not specified	Not specified	Not specified	N/Not specifiA	NNot specified/A	NNot specified/A	Information overload, fatigue and many other factors medical image perception.
[42]	Kim et al. (2014)	USA	Retrospective cohort study	656	Radiologic examinations with delayed diagnoses	Not specified	Radiologic examinations	Effected of errors on delayed diagnosis	Diagnostic errors	In one third of cases, delayed diagnoses were not recognized on subsequent radiologic examinations

### 3.2. Diagnostic Limitations by Neurotologic Lesion

A summary of imaging performance, key limitations, and clinical implications across neurotologic lesion subtypes is provided in Table 2.

**Table 2:** A summary of imaging performance and clinical implications by neurotologic lesion.

Article Reference	First Author (Year)	Lesion Type	Imaging Modality	Reported Sensitivity (%)	Reported Specificity (%)	Major Limitation	Clinical Implications
[10,16]	Piekarek et al. (2022); Pietraszek et al. (2025)	Cholesteatoma	Non-EPI DWI	87–100	83.3	Requires clinical correlation and follow-up imaging	Preferred technique for detection of primary and recurrent cholesteatoma
[21]	Forgues et al. (2018)	Vestibular Schwannoma	HRT2-WI	77.8	88.2	Less structural detail than contrast-enhanced MRI	Useful for initial diagnosis; complementary to Gd T1C-MRI
[22]	de Bresser et al. (2024)	Paraganglioma	68Ga PET/CT	93	85	Limited availability and higher cost	Preferred modality for lesion detection, exclusion of mimics, and assessment of multifocal or metastatic disease
[25]	Kim et al. (2023)	NOE	Tc-99; Ga-67	97 93.8	NR NR	Limited anatomic resolution; Poor spatial resolution	Highly sensitive for early diagnosis and monitoring of disease progression
[29]	Berrettini et al. (2010)	Otosclerosis	SPECT	95.2	96.7	Limited anatomic detail	Superior differentiation of active otosclerotic bone; useful when HRCT findings are equivocal

*NR, not reported.*

#### 3.2.1. Cholesteatomas

EP-DWI is a reliable modality for the detection of cholesteatoma compared with high-resolution computed tomography (HRCT) [15]. EP-DWI demonstrated higher sensitivity (88.4% vs. 69%), specificity (92.8% vs. 67.8%), positive predictive value (92% vs. 66.6%), and negative predictive values (89.6% vs. 73.07%) compared with HRCT. Overall, EP-DWI correctly identified the presence or absence of cholesteatoma in 90.7% of patients, whereas HRCT was accurate in 68.5% [15].

Non-EPI DWI has emerged as the preferred DWI technique for cholesteatoma detection, with a reported sensitivity of 87% [16]. However, Pietraszek et al. recommend thorough clinical correlation and repetitive scanning to monitor for recurrence and to reduce the false-positive (8%) and false-negative (12%) findings [16]. Compared with echo-planar imaging (EPI DWI), non-EPI DWI demonstrated higher reported sensitivity compared with EPI DWI in this cohort (100% vs. 69.2%), specificity (83.3% vs. 66.6–83.3%), positive predictive value (96.3% vs. 90.0–94.7%), and negative predictive value (100% vs. 33.3–38.4%) [10].

Several studies emphasized the value of combining DWI with CT for optimal imaging and diagnosis of cholesteatoma. Fused images (DWI-CT) demonstrated higher sensitivity (57.1% vs. 52.1%), specificity (94.8% vs. 88.9%), and accuracy (81.8% vs. 75.8%) compared with DWI alone [11]. Locketz et al. further showed that combining Periodically Rotated Overlapping Parallel Lines with Enhanced Reconstruction (PROPELLER) diffusion-weighted magnetic resonance imaging (DW-MRI) with corresponding temporal bone computed tomography (CT) increased localization accuracy to 90%, compared with PROPELLER DW-MRI alone (83%) or CT alone (62.5%) [17].

#### 3.2.2. Vestibular Schwannoma (Acoustic Neuroma)

Gadolinium-enhanced T1-weighted MRI (Gd T1C-MRI) is commonly used as a first-line imaging modality for diagnosis of vestibular schwannoma [18,19]. It is particularly useful for excluding alternative diagnoses and for evaluation of patients following surgical or radiosurgical intervention, where postoperative anatomical alterations may obscure landmarks. Three-dimensional High-Resolution T2-WI (HRT2-WI) MRI offers excellent spatial resolution and is currently indicated for initial diagnosis of vestibular schwannoma [18]; however, it provides less structural detail compared with Gd T1C-MRI. Nonetheless, small intracanalicular tumors and postsurgical recurrence remain a source of false negatives across both T1- and T2- weighed MRI sequences.

Coelho et al. found no statistically significant differences in tumor measurements between Gd T1C-MRI and HRT2 sequences across all imaging planes [19], and Tolisano et al. reported no significant inter-reviewer variation in lesion diameters or volumes between the two techniques [20]. Nonetheless, Gd T1C-MRI demonstrated superior accuracy in assessing tumor growth over time. In a separate study, Forgues et al. reported a specificity of 88.2% and a sensitivity of 77.8% for T2-weighted MRI in the detection of vestibular schwannomas, underscoring both the reliability of T2 imaging and the complementary value of contrast-enhanced MRI for comprehensive lesion assessment [21].

#### 3.2.3. Glomus Tumors (Paragangliomas)

CT and MRI are the current standard imaging methods in the diagnosis of paragangliomas. However, 68Ga-DOTATOC PET/CT has emerged as a promising technique for detecting head and neck paragangliomas [22]. This modality complements CT and MRI by more reliably excluding skull base mimics and narrowing the differential to neuroendocrine tumors, facilitating tailored management with less invasive procedures. 68Ga-DOTATOC PET/CT is also the preferred imaging method for excluding metastatic or multifocal disease [23].

Lesion-based detection rates are higher for 68Ga-DOTATOC PET/CT (98.6%) compared with CT/MRI (85.8%) [22]. Reported pooled sensitivity and specificity are 93% and 85%, respectively. Additionally, a retrospective analysis comparing the performance of 68Ga-DOTATATE PET/CT and 123I-MIBG SPECT concluded that 68Ga-DOTATATE PET/CT should be considered as the first-line choice in patients with a high probability of paragangliomas [24].

#### 3.2.4. Malignant (Necrotizing) Otitis Externa

Kim et al. conducted a comparative analysis of multiple imaging modalities for necrotizing otitis externa (NOE), including Gallium-67 and Technetium-99m scintigraphy, MRI, and CT [26]. The pooled sensitivities were highest for Technetium-99m and Gallium-67 at 97% and 93.8%, respectively, followed by CT (94%) and MRI (71%) [25]. These findings indicate that CT remains a valuable tool in the diagnosis of NOE, particularly for assessing bony erosion. However, CT may yield false-negative results during early disease stages when bone involvement is minimal.

Nuclear medicine techniques, particularly Gallium-67 and Technetium-99m scintigraphy, have demonstrated superior sensitivity for both initial diagnosis and monitoring of disease progression. In a recent study, Haleem et al. reported that CT identified findings consistent with NOE in 64% of cases, whereas Technetium-99m scintigraphy detected 56% additional positive findings among patients with CT-negative scans [26]. These results exemplify the complementary role of nuclear medicine in improving diagnostic sensitivity, particularly when CT findings are inconclusive or when early detection is critical to guide management.

#### 3.2.5. Otosclerosis

HRCT remains the imaging method of choice for the evaluation of otosclerosis, providing superior delineation of bony anatomy and precise detection of both fenestral and retro-fenestral foci. MRI serves as a valuable adjunct in patients who present with sensorineural or mixed hearing loss, as it enables assessment of cochlear lumen patency and potential cochlear involvement, particularly in patients with advanced disease who might require cochlear implantation [27].

In a retrospective study of HRCT diagnostic accuracy, Bassiouni et al. reported a “substantial rate of missed diagnoses in clinical practice,” with general radiologists failing to identify 45.4% of cases that were subsequently detected by subspecialized neuroradiologists [28]. This study underscores the importance of expertise in temporal bone imaging interpretation.

Comparative studies have explored the diagnostic performance of HRCT vs. single-photon emission computed tomography (SPECT) in detecting otosclerosis. Berrettini et al. reported a sensitivity of 95.2% and specificity of 96.7% for SPECT, markedly higher than the 58% sensitivity observed with HRCT [29]. These findings suggest that SPECT demonstrated higher reported sensitivity and specificity compared with HRCT in this study between normal and pathologic bone, potentially enhancing diagnostic accuracy in cases where HRCT findings are correlative [29]. Interpretation of comparative performance should be made cautiously due to heterogeneity in study design, outcome definitions, and reader expertise across included studies.

### 3.3. Socioeconomic and Geographic Barriers

Socioeconomic and geographic factors directly impact access to advanced neuroimaging services. Vagal et al. (2017) demonstrated that older adults, Black patients, and rural residents consistently had lower utilization of CTA, MRI, and MRA, highlighting demographic disparities in acute stroke imaging [30]. Wang et al. (2024) similarly reported gender- and race-based differences, with rural women, Black patients, and individuals over 80 years old having reduced imaging utilization, reinforcing patterns of inequity across multiple patient subgroups [31]. In addition, Wang et al. (2022) found that patients from lower socioeconomic subgroups were significantly less likely to receive advanced neuroimaging and timely acute interventions, emphasizing that economic disadvantage limits access to diagnostic pathways and delays treatment [32].

Evidence from low-income countries further reflect these disparities, as in a multicenter In a Pakistani study, Khalid et al. reported that over half of patients with intracranial schwannomas belonged to low socioeconomic subgroups [33]. Nearly 78% were treated in public hospitals, with over 40% lost to follow-up. This data highlights how scarce financial resources and dependence on public-sector facilities restrict both imaging access and continuity of care. Insurance status pushes these disparities even further, as uninsured, Medicaid, and Medicare patients had significantly lower odds of undergoing noninvasive head and neck angiography and MRI compared to privately insured patients [34]. Household income is another determinant, with patients from census tracts with lower median household incomes being less likely to receive advanced neuroimaging such as MRI and MR angiography (MRA) [35].

Geographic variability has also been observed, with higher CT angiography (CTA) and CT perfusion (CTP) utilization in the Northeastern and Western US compared to the Southern Stroke Belt, where reliance on MRI and MRA was greater despite their lower sensitivity for posterior fossa lesions [35]. Zeitouni et al. demonstrated that specific zip codes are significantly more likely to have their imaging complete prior to otolaryngology appointments (*p* = 0.004) [36]. Beyond underuse, Wang et al. also highlights patterns of selective overuse, as white, privately insured, higher-income, middle-aged male patients were disproportionately more likely to undergo CTA, particularly post guideline updates, suggesting implicit bias [31].

Demographic patterns in vestibular schwannomas reflect similar disparities in disease detection and management. Carlson et al. (2016) reported racial differences in vestibular schwannoma incidence, noting that Black patients were underrepresented in large clinical cohorts, suggesting potential disparities in diagnosis or referral patterns [37]. Pandrangi et al. (2020) analyzed contemporary management trends, highlighting increased use of conservative management strategies and emphasizing the importance of individualized care based on tumor size, growth rate, and patient comorbidities [38]. These studies underscore that both disease prevalence and clinical decision-making are shaped by patient demographics and access to specialized care.

### 3.4. Interpretation Bias and Expertise Variability

Interpretation bias refers to both perceptual errors (failure to detect abnormalities) and cognitive errors (misclassification of detected findings), which are influenced by reader experience, fatigue, workload, and clinical context. Mullins et al. demonstrated that providing clinical suspicion of stroke increased CT sensitivity from 38% to 52%, highlighting the powerful influence of diagnostic framing on perception [39]. Inter-observer variability further contributes to diagnostic inconsistency, with Oray et al. reporting only moderate agreement (κ = 0.60–0.67) among readers interpreting diffusion-weighted MRI for acute ischemia [40]. These findings emphasize that diagnostic performance is not solely modality-dependent but strongly shaped by human factors and reader expertise. Radiologic interpretation is ultimately a human enterprise dependent on complex physiologic and cognitive processes. It is unsurprisingly subject to a wide range of error types, such as perceptual errors, where abnormalities are not seen, and cognitive errors, where detected findings are not correctly understood or appreciated. Mullins et al. highlighted that interpretation is shaped strongly by context, as the sensitivity of non-contrast CT for acute infarction rose from 38% to 52% when clinical suspicion was provided, validating expectations’ influence on perception [39]. This reliance on clinical framing reflects a broader issue of interpretation bias, in which subtle posterior fossa or neurologic lesions may be overlooked or dismissed as migraine or anxiety when not anticipated.

Inter-observer variability further complicates diagnosis. In diffusion-weighted MRI, agreement between readers was only moderate to substantial (κ = 0.60–0.67) with notable rates of false positives and false negatives, demonstrating that even high-yield modalities are not immune to inconsistency [40]. Expertise also plays a major role, as inexperienced readers misclassified posterior fossa ischemia and small infarcts more frequently, whereas consensus review by senior neuroradiologists corrected 25–40% of ambiguous cases, reflecting the impact of specialized training and experience [41].

Variability also stems from technical and human factors, including differences in MRI devices, display conditions, fatigue and individual perceptual thresholds [41]. The clinical consequences are significant, with perceptual misses accounting for up to 30% of diagnostic errors in radiology, and interpretive disagreements affect treatment decisions directly [42]. False positives can cause unnecessary clinical intervention, while false negatives can cause delay in clinical intervention, allowing progression into much larger and more malignant lesions. Although radiologist expertise is central to accurate interpretation, relatively fewer large-scale studies categorize imaging performance by reader experience, which limits the generalizability of reported diagnostic sensitivity across different settings. Although a structured Boolean search strategy was employed, no search approach is fully exhaustive. Relevant studies may have been missed due to variations in terminology, database indexing practices, or publication bias.

## 4. Discussion

Advances in neuroimaging have substantially improved the diagnosis and treatment of neurotologic lesions. The high sensitivities and specificities now achievable across many imaging modalities underscore how far the field has progressed since the introduction of these technologies. Yet despite these gains, diagnostic uncertainty remains a persistent challenge. Limitations still exist for every modality. Small lesions may elude detection, artifacts may imitate pathology, and the complex interface of bone and neurovascular structures continues to resist perfect visualization.

Beyond the intrinsic capabilities of the imaging tools, a more pressing issue lies in how these modalities are applied in practice. Diagnostic performance varies across patient populations, influenced by age, sex, race, comorbidities, and socioeconomic status. Individuals in rural or socioeconomically disadvantaged settings are more likely to receive lower-yield imaging, increasing the risk of missed diagnoses or misclassification as migraine, anxiety, or functional symptoms [43]. Conversely, selective overuse in higher-resourced groups may lead to unnecessary costs and radiation exposure without corresponding improvements in outcomes [44]. These disparities in imaging access contribute directly to disparities in long-term clinical outcomes [45]. Patients without access to high-yield modalities such as non-EPI DWI or contrast-enhanced MRI may experience delayed diagnoses, increased tumor growth, and worsened prognoses.

A major gap in the literature is the underrepresentation of low-income and rural populations. Only a small number of studies stratify diagnostic performance by socioeconomic or geographic context, and even fewer examine these patterns specifically in neurotologic disease. Large prospective cohorts and machine-learning models frequently omit stratification by race, sex, and socioeconomic status. As a result, patients who already face barriers to care may also face disproportionate rates of missed or delayed diagnoses [45]. In order to address these gaps, future work must incorporate validation across diverse patient populations, standardized reporting of imaging use across socioeconomic and regional groups, and policies that ensure equitable access to high-yield modalities.

As technologies continue to evolve, current approaches to diagnosing and treating neurotologic lesions must expand in parallel, particularly with the emergence of artificial intelligence-assisted imaging, advanced MRI sequences, and hybrid PET/MRI platforms. Machine learning models have demonstrated promise in lesion detection and risk stratification; however, multiple studies have shown performance degradation when models are applied across institutions with differing patient demographics and imaging protocols, underscoring the need for local validation. However, these technological improvements must occur in lockstep with addressing the existing barriers to equitable access. Further progress in neurotologic imaging will hinge on the fairness of its application, where diagnostic clarity is no longer contingent on background or circumstance.

The evidence base is highly heterogeneous, with substantial variability in study design, patient populations, imaging protocols, outcome definitions, and radiologist expertise. This heterogeneity limits direct cross-study comparisons and precludes definitive modality-to-modality superiority claims. Accordingly, comparative language throughout this review has been intentionally restrained unless supported by direct head-to-head data. Finally, we synthesized available data narratively rather than meta-analytically where clinical heterogeneity was substantial, which may limit precision but preserves interpretability across diverse contexts.

This review is inherently limited by the search approach employed, in that relevant articles may have been potentially missed if they were not identified by the string. Further, given the rapidity of new developments in neurotological imaging, results should be interpreted within the specific time frame given.

## 5. Conclusions

Neuroimaging plays a central role in the diagnosis of neurotologic lesions, yet important limitations remain. Diagnostic accuracy varies by imaging modality, lesion type, and disease stage. Small or early lesions are particularly prone to being missed, even with advanced MRI and CT protocols. These technical constraints are further affected by interpretation bias and variability in radiologist expertise.

This review demonstrates that neuroimaging performance is not uniform across patient populations. Access to high-yield imaging is strongly influenced by socioeconomic status, geographic location, and insurance coverage. Patients from underserved or rural communities are more likely to experience delayed diagnosis or misclassification, while selective overuse is observed in higher-resourced populations. These disparities contribute directly to unequal clinical outcomes.

Improving diagnostic accuracy will require more than incremental technological advances. Future research should include large, prospective studies that stratify imaging performance by patient characteristics and reader expertise. Standardized reporting and validation across diverse populations are essential. Emerging tools, including advanced imaging techniques and artificial intelligence-assisted interpretation, may reduce variability, but only if paired with equitable access to care. Addressing both technical and structural barriers is critical to improving outcomes in neurotologic disease.

## Data Availability

The data supporting this review’s findings can be obtained from the corresponding authors upon request.

## Supplementary Materials

The following supporting information can be downloaded at: https://www.mdpi.com/article/10.3390/diagnostics16030446/s1. **Table S1**. PRISMA 2020 Checklist for the Systematic Review. Source: PRISMA 2020 checklist (CC BY 4.0). https://www.prisma-statement.org/prisma-2020-checklist (accessed on 26 January 2026). Reference [46] is cited in the supplementary materials.

## Abbreviations

The following abbreviations are used in this manuscript:

CT	Computed Tomography
CTA	Computed Tomography Angiography
CTP	Computed Tomography Perfusion
DWI	Diffusion-Weighted Imaging
EP-DWI	Echo-Planar Diffusion-Weighted Imaging
FLAIR	Fluid-Attenuated Inversion Recovery
Gd T1C-MRI	Gadolinium-Enhanced T1-Weighted Magnetic Resonance Imaging
HRCT	High-Resolution Computed Tomography
HRT2-WI	High-Resolution T2-Weighted Imaging
MRI	Magnetic Resonance Imaging
MRA	Magnetic Resonance Angiography
NOE	Necrotizing Otitis Externa
Non-EPI DWI PET	Non-echoplanar diffusion-weighted imaging Positron Emission Tomography
PRISMA	Preferred Reporting Items for Systematic Reviews and Meta-Analyses
PROPELLER	Periodically Rotated Overlapping Parallel Lines with Enhanced Reconstruction
SPECT	Single Photon Emission Computed Tomography
US	United States
VS	Versus
